# Enhanced Performance of Carbon–Selenide Composite with La_0.9_Ce_0.1_NiO_3_ Perovskite Oxide for Outstanding Counter Electrodes in Platinum-Free Dye-Sensitized Solar Cells

**DOI:** 10.3390/nano12060961

**Published:** 2022-03-14

**Authors:** Arnauld Robert Tapa, Wanchun Xiang, Senwei Wu, Bin Li, Qiufen Liu, Mingfeng Zhang, Marzieh Ghadamyari, Francis Verpoort, Jichao Wang, Albert Trokourey, Xiujian Zhao

**Affiliations:** 1 State Key Laboratory of Silicate Materials for Architecture, Wuhan University of Technology, Luoshi Road, Wuhan 430070, China; tapaarnauld@yahoo.fr (A.R.T.); senwei_wu@whut.edu.cn (S.W.); libin625@whut.edu.cn (B.L.); qiufen.liu@whut.edu.cn (Q.L.); 15084978457@163.com (M.Z.); 2 Laboratory of Constitution and Reaction of Matter, Training and Research Unit for Structural Sciences of Matter and Technology, Félix Houphouët-Boigny University of Cocody-Abidjan, Abidjan 22 BP 582, Côte d’Ivoire; trokourey@gmail.com; 3 Key Laboratory for Applied Surface and Colloid Chemistry, Ministry of Education, Shaanxi Key Laboratory for Advanced Energy Devices, Shaanxi Engineering Laboratory for Advanced Energy Technology, School of Materials Science & Engineering, Shaanxi Normal University, Xi’an 710119, China; 4 Laboratory of Organometallics, Catalysis and Ordered Materials, State Key Laboratory of Advanced Technology for Materials Synthesis and Processing, Wuhan University of Technology, Wuhan 430070, China; marzieh.ghadamyari@yahoo.com (M.G.); francis.verpoort@ghent.ac.kr (F.V.); wangjichao5475@163.com (J.W.); 5 National Research Tomsk Polytechnic University, Lenin Avenue 30, 634050 Tomsk, Russia

**Keywords:** counter electrode, perovskite oxide, carbon materials, metal selenides, metal-organic-frameworks

## Abstract

For large-scale applications, dye-sensitized solar cells (DSSCs) require the replacement of the scarce platinum (Pt)-based counter electrode (CE) with efficient and cheap alternatives. In this respect, low-cost perovskite oxides (ABO_3_) have been introduced as promising additives to composite-based CEs in Pt-free DSSCs. Herein, we synthesized composites from La_0.9_Ce_0.1_NiO_3_ (L) perovskite oxide and functionalized-multiwall-carbon-nanotubes wrapped in selenides derived from metal-organic-frameworks (f-MWCNT-ZnSe-CoSe_2_, “F”). L and F were then mixed with carbon black (CB) in different mass ratios to prepare L@CB, F@CB, and L@F@CB composites. The electrochemical analysis revealed that the L@F@CB composite with a mass ratio of 1.5:3:1.5 exhibits better electrocatalytic activity than Pt. In addition, the related DSSC reached a better PCE of 7.49% compared to its Pt-based counterpart (7.09%). This improved performance is the result of the increase in the oxygen vacancy by L due to the replacement of La with Ce in its structure, leading to more active sites in the L@F@CB composites. Moreover, the F@CB composite favors the contribution to the high electrical conductivity of the hybrid carbon nanotube–carbon black, which also offers good stability to the L@F@CB CE by not showing any obvious change in morphology and peak-to-peak separation even after 100 cyclic voltammetry cycles. Consequently, the corresponding L@F@CB-based device achieved enhanced stability. Our work demonstrates that L@F@CB composites with a low cost are excellent alternatives to Pt CE in DSSCs.

## 1. Introduction

Dye-sensitized solar cells (DSSCs) are one of the third-generation photovoltaic technologies that have acquired extensive consideration because of their cheap manufacturing cost and potential to reach a power conversion efficiency (PCE) of over 14% up to now [1]. Generally, a DSSC is comprised of four principal parts, namely a photoanode, a dye-sensitizer, an electrolyte containing a redox mediator, and a counter electrode (CE) [2]. The CE plays an important role in the design of DSSCs as it eases the reduction of redox species by a continuous production of electricity and the transfer of electrons from the outer circuit back to the electrolyte [3]. Platinum (Pt) is commonly used as the CE for DSSCs thanks to its superior electrocatalytic activity and high charge-transfer capability. However, the high cost of Pt is an impediment to the development of DSSCs. This led to the development of research directed toward the production of cheap, stable, and efficient alternatives to Pt CEs in DSSCs. In the past, different alternative materials to Pt, such as chalcogenides, carbon-based materials, metal oxides, etc., were explored in DSSCs [4,5,6,7,8].

Perovskite oxides (ABO_3_) are a special type of metal oxide that has attracted attention thanks to their compositional, structural flexibility, and fascinating electrocatalytic properties. Furthermore, their physicochemical properties and catalytic activity can be controlled by doping components at various positions $A_{x}A_{1-\;x}^{\prime }B_{y}B_{1-\;y}^{\prime }O_{3}$ [9,10,11,12,13]. However, very little research has been conducted so far on the use of perovskite oxides as CEs in DSSCs, probably because of their low electrical conductivity, small surface areas, and low PCEs observed when used solely [14]. LaNiO_3_ is a simple and low-cost perovskite oxide that has drawn considerable attention in various fields thanks to its chemical stability, relatively high conductivity compared to other perovskite oxides, and also because it presents one of the highest oxygen reduction reaction activities reported for this kind of perovskite oxide [15,16,17,18]. Recently, Yu et al. [19] demonstrated that partial replacement of La sites with Ce improves the performance of LaNiO_3_ as oxygen evolution reaction catalysts by increasing the O 2p band center. This results in an appropriate amount of oxygen vacancy and enhanced structural flexibility. Therefore, they adopted the glycine–nitrate precursor (GNP) method to prepare La_0.9_Ce_0.1_NiO_3_ (L) perovskite oxide. In the process, glycine was used as a reducing and pore-generating agent, resulting in a mesoporous and large-surface-area perovskite oxide material [20]. As the oxygen vacancies in some simple metal oxides are one of the principal contributors to catalyzing $I_{3}^{-}$ reduction reactions (IRR) in DSSCs, plenty of oxygen vacancies in the perovskite oxide lattice lead to more active sites which minimize efficiency losses in DSSCs [17,21].

Furthermore, it was demonstrated that adding perovskite oxides to carbon-based materials may combine their properties for efficient Pt-free DSSCs. With this regard, Xiong et al. [22] prepared, via a physical-mixing method, the first perovskite oxide/carbon (La_0.65_Sr_0.35_MnO_3_/graphene, “LSMO@RGO”) composite for CE application in Pt-free DSSCs. The PCE of 4.93% achieved for RGO significantly increased to 6.57% for LSMO@RGO-8%, which unfortunately showed a lower value than Pt-based DSSCs (7.13%). However, this promising result confirmed that adding perovskite oxides to carbon-based materials by a simple physical-mixing method allows improvement of the electrocatalytic activity of CEs in DSSCs.

Although the perovskite oxide–carbon composites present some benefits for DSSC applications, the choice of the carbon material remains important for better performance. Among carbon-based materials, carbon nanotubes (CNT) have drawn extraordinary interest thanks to their exceptional structure and outstanding performance in Pt-free DSSCs [23,24]. However, when used alone as a CE for DSSCs, CNT suffers from low adhesion between the carbon film and the substrate, which affects the stability of this carbon-based CE in DSSCs. Therefore, carbon–carbon composites were explored, and it was proven that combining CNT with other carbon-based materials such as CB could significantly improve their conductivity, electrocatalytic activity, and stability for application as CEs in DSSCs. This is possible thanks to the high electrical conductivity, and electrocatalytic activity of CB, which are added to its strong binding capability with FTO substrates [25]. Indeed, Cruz-Gutiérrez et al. [25] successfully demonstrated that the hybrid CNT@CB composite-based CE is better than the corresponding CNT and CB-based counterparts in DSSCs. However, no research work has been reported so far on the use of this hybrid with perovskite oxide in DSSCs, probably due to the low performance reported for CNT and CB when used separately as CE in DSSCs. Incorporating perovskite oxides into the hybrid CNT@CB could add to their high electrical conductivity, more active sites, and the possibility to increase the oxygen vacancies as mentioned earlier [17,26].

Moreover, it was reported that wrapping carbon-based materials with selenide compounds derived from metal-organic-frameworks (MOFs) allows the achievement of highly porous materials suitable for CE in DSSCs, thanks to the regular nanostructured pores and high surface area of MOFs [27,28]. Jian et al. [28] synthesized a zinc selenide wrapped in N-doped carbonaceous (ZIF-ZnSe-NC) for use as CE for DSSCs. This ZIF-ZnSe-NC was prepared via carbonization and selenization of ZIF-7. A PCE (8.69%) higher than that of the DSSC with Pt CE (8.26%) was achieved for the DSSC designed with ZIF-ZnSe-NC-11 wt% CE. Unfortunately, these composites have also not been explored yet with perovskite oxides, which could minimize the efficiency losses in DSSCs by offering more active sites [17,21].

In this study, we synthesized composites from L perovskite oxide obtained from the GNP method and F prepared from the hydrothermal selenization of functionalized-multiwall-carbon-nanotubes wrapped in zeolitic imidazolate frameworks (f-MWCNT/ZIF-8@ZIF-67). The L perovskite oxide was then used as an additive to F and CB to prepare composites, and the performance as CEs for DSSCs was observed. By optimizing the mass ratios among L, F, and CB in L@F@CB composites, we are able to obtain better electrocatalytic activity than Pt. Furthermore, DSSCs based on L@F@CB composite achieved a higher PCE of 7.49% in comparison to the PCE of their Pt-based DSSC (7.09%) counterpart. This work demonstrates that L can increase the oxygen vacancies in L@F@CB composites for more active sites and better CEs in DSSCs. This gives rise to more active sites in the final L@F@CB composite for better CEs in DSSCs. Therefore, the corresponding L@F@CB-based device achieved enhanced stability.

## 2. Experimentation Section

### 2.1. Materials and Reagents

MWCNT, lithium perchlorate trihydrate (LiClO_4_·3H_2_O), lithium iodide (LiI), silver nitrate (AgNO_3_), aqueous ammonia solution (NH_4_OH), titanium diisopropoxide bis (acetylacetonate) (TAA)[(CH_3_)_2_CHO]_2_Ti(C_5_H_7_O_2_)_2_, 75 wt. % in isopropanol], iodine (I_2_), platinum chloride (H_2_PtCl_6_), 4-tert-butylpyridine (4-tBP), disodium ethylene diamine tetraacetate dihydrate (EDTA–2Na), glycine, polyethylene glycol-20000 (PEG-20000), and acetonitrile were purchased from Sigma Aldrich, Beijing, China. Cis-Di(thiocyanato)bis(2,2′-bipyridyl-4,4′-dicarboxylate) ruthenium (II) (N719) was bought from Dyesol, Queanbeyan, Australia. Nickel *nitrate* hexahydrate (Ni(NO_3_)_2_·6H_2_O), lanthanum (III) nitrate hexahydrate (La(NO_3_)_3_·6H_2_O), cerium *nitrate* hexahydrate (Ce(NO_3_)_3_·6H_2_O), selenium powder (Se, >99.99% metal basis), sodium hydroxide (NaOH), zinc nitrate hexahydrate (Zn(NO_3_)_2_·6H_2_O), 2-methylimidazole (MeIm), and cobalt chloride hexahydrate (CoCl_2_·6H_2_O) were obtained from Aladdin, Beijing, China. Titanium dioxide paste and FTO conductive glass (2 mm thickness, sheet resistance 10–15 Ω. sq^−1^) were purchased from OPV Tech Co., Yingkou, Liaoning, China.

### 2.2. Synthesis of L

The synthesis of L perovskite oxide nanoparticles was performed following previous literature, by a hydrothermal synthesis method using a GNP [19,20]. First, 20 mmol of La(NO_3_)_3_·6H_2_O (8.6602 g), 2.2 mmol of Ce(NO_3_)_3_·6H_2_O (0.9553 g), and 22 mmol of Ni(NO_3_)_2_·6H_2_O (6.3976 g) were added to 50 mL of DI water and stirred for 15 min. Then, 110.5 mmol of aqueous glycine (8.2952 g) was added dropwise to the mixture solution and stirred for 10 min. The mixture was finally adjusted to a pH of 7.7 with 1 M NH_4_OH and stirred for an additional 10 min. When the pH was adjusted, the color of the mixture changed from pale green to an intense blue. Finally, we transferred the mixture into a Teflon-lined autoclave and heated it for 12 h at 180 °C. The obtained pale blue precipitate was washed with water and ethanol several times for 10 min each time, then dried at 80 °C for 12 h. After grinding the product into powder, it was calcinated to 700 °C at 5 °C·min^−1^ for 2 h in an air atmosphere oven.

### 2.3. Functionalization of MWCNT

The functionalization of MWCNT was performed according to the previous report [29]. In brief, 1.5 g of raw MWCNT was refluxed in a mixture solution of HNO_3_/H_2_SO_4_ combined in a 1:3 ratio to create a solution with a final volume of 50 mL. The above-mixed solution was treated by vigorous magnetic stirring at 70 °C for 12 h. The resulting product was cooled and centrifuged at 4000 rpm for 30 min, followed by successive washings with distilled water and ethanol at 4000 rpm for 10 min each time. The final product was dried at 90 °C and named *f*-MWCNT.

### 2.4. Preparation of f-MWCNT/ZIF-8@ZIF-67 Composite

We synthesized *f*-MWCNT/ZIF-8@ZIF-67 by a little modification of a ZIF-8 seed-mediated approach used in previous reports [27]. In short, we first prepared *f*-MWCNT/ZIF-8 via the deposition of ZIF-8 seeds on *f*-MWCNT. In brief, we mixed two methanolic solutions of Zn(NO_3_)_2_·6H_2_O (4.545 g, 20 mL) and MeIm (6.818 g, 20 mL) under stirring. Then, we added to the above mixture 20 mL of *f*-MWCNT solution (5 mg. mL^−1^, in water/methanol, 1:4, *v/v*). After about 30 min of stirring, we transferred the mixture into a Teflon-lined autoclave and kept it at 100 °C for 12 h. A formed grey solid was obtained via centrifugation (8000 rpm, 10 min) after cooling to room temperature and washed at least 3 times with methanol. *f*-MWCNT/ZIF-8 was obtained after 12 h drying at 80 °C. In the following step, ZIF-67 was deposited onto *f*-MWCNT/ZIF-8. In brief, we mixed under stirring methanolic solutions of *f*-MWCNT/ZIF-8 (1.010 g, 20 mL), cobalt chloride hexahydrate (CoCl_2_.6H_2_O) (2.209 g, 20 mL), and MeIm (11.248 g, 20 mL). After 30 min of stirring for homogenization, we kept the mixture at room temperature for 12 h. After several centrifugations (8000 rpm, 10 min) and washed with methanol, the resulting product (*f*-MWCNT/ZIF-8@ZIF-67) was collected and dried at 80 °C for 12 h.

### 2.5. Preparation of f-MWCNT-ZnSe-CoSe_2_

For the preparation of *f*-MWCNT-ZnSe-CoSe_2_, we performed the selenization of *f*-MWCNT/ZIF-8@ZIF-67 composite based on a previously reported hydrothermal synthesis of CoSe_2_. [27] First, we prepared a selenium (Se) precursor solution by dissolving NaOH (19.2837 g) in 30 mL of deionized (DI) water, then selenium powder Se (1.5427 g) was added and completely dissolved by heating the mixture at 80 °C. Secondly, we dissolved *f*-MWCNT/ZIF-8@ZIF-67 powder (2.3234 g) in an EDTA–2Na aqueous (aq.) solution (10 mL, 80 mM). After heating at the same temperature both precursor solutions of *f*-MWCNT/ZIF-8@ZIF-67 and Se, we mixed them, then stirred while heating the mixture for 10 min. Finally, we transferred the mixture into a Teflon-lined autoclave for 12 h heating at 170°C. After several centrifugations (8000 rpm, 10 min), and washing with water and ethanol of the cooled mixture, a black precipitate of the *f*-MWCNT-ZnSe-CoSe_2_ composite was obtained, dried for 12 h at 60 °C, and named F.

### 2.6. Counter Electrodes’ Preparation

For the preparation of F, L, and CB-based CEs, 0.06 g of each compound was separately mixed with 0.02 g of PEG-20000, and then we added 4 mL of ethanol while milling in a mortar to form a paste. As for the preparation of L@F@CB composites based on CEs, we prepared 0.06 g in the mass ratios of 1:4:1, 1.5:3:1.5, and 2:2:2 of L: F: CB in 0.02 g of PEG-20000, corresponding respectively to 1-L@F@CB, 2-L@F@CB, and 3-L@F@CB. Then, each dispersion was spin-coated onto FTO glass substrates and dried for 20 min at 120 °C, followed by heating at 200 °C for 30 min. We also prepared Pt CEs for comparison via the thermal decomposition of H_2_PtCl_6_ (10 mM) in ethanol at 380 °C followed by the drop-casting of the Pt solution onto FTO glass and heating at 400 °C for 20 min [30].

### 2.7. Fabrication of TiO_2_ Photoanode and Full DSSCs

Based on previous literature, we prepared TiO_2_ pastes [31]. First, we washed and rinsed FTO glass substrates in several ultrasonic baths for 15 min each, using a water–detergent solution, deionized water, and ethanol, respectively. Then, the cleaned FTO glass was heated to 450 °C on a hot plate, and we sprayed by airbrush a 10% (*v/v*) alcoholic solution of titanium isopropoxide onto its conductive side, to form a blocking compact layer. This was followed by the screen printing onto the blocking layer of commercial TiO_2_ pastes. In brief, the working electrode was made of 5 layers of a transparent TiO_2_ paste (18 nm) coated on top of the blocking layer, and 3 layers of scattering TiO_2_ paste (400 nm) coated on top of the transparent TiO_2_ layers. Then the electrode was annealed at 120 °C for 10 min each time after the printing of each layer of TiO_2_ paste. The final TiO_2_ films having an active area of 0.16 cm^2^ were heated under an airflow oven system by progressive sintering at 325 °C for 5 min, 375 °C for 5 min, 450 °C for 15 min, and 500 °C for 30 min, respectively, and cooled in air at room temperature. After cooling, the electrode was TiCl_4_ treated at 70 °C for 30 min in a TiCl_4_ (aq.) (20 Mm) bath, then rinsed in deionized water and ethanol, respectively. Before dye loading, we heated the TiO_2_ electrode at 500 °C for 30 min, cooled it, and immersed it for 24 h in a dye solution of N719 (0.3 mM). We assembled the DSSCs by clipping the sensitized TiO_2_ photoanode onto a prepared CE. The photoanode and CE were separated by a plastic joint spacer of 25 µm thickness and an inner dimension of 5 × 6 mm. Then, the liquid electrolyte was injected into the gap between the photoanode and the CE.

### 2.8. Characterization Methods

X-ray diffraction (XRD) was employed to analyze the structure of our compounds. The measurements were made on a Bruker D8 advance X-ray diffractometer with monochromatized Cu Kα radiation. The chemical states and nature of chemical bonds were analyzed via X-ray photoelectron spectroscopy (XPS), measured at a base pressure of <10^−9^ Torr on a VG Multi-Lab 2000 spectrometer. To investigate the surface area and pore size distribution of the prepared nanoparticle powders, we employed the N_2_ gas adsorption/desorption method on a Micromeritics ASAP 2020 HD88 system. The surface area was measured using the Brunauer–Emmett–Teller (BET) method, while the pore size distribution was measured using the Barrett–Joyner–Halenda (BJH) method. A field emission scanning electron microscope (FESEM, JSM 7100F), and a high-resolution transmission electron microscope (HRTEM, JEM-2100F) using an energy dispersive spectrum (EDS) analyzer were used to examine the morphology of the different CEs. For the electrochemical analysis, we used a CHI760D electrochemical workstation to perform cyclic voltammetry (CV), electrochemical impedance spectroscopy (EIS), and Tafel polarization plot measurements. The CV system was made of a reference electrode Ag/Ag^+^ (10 mM AgNO_3_ in acetonitrile), a Pt wire counter electrode, and a working electrode composed of the CE to be tested. The three electrodes were immersed in an acetonitrile electrolyte solution of 0.1 M LiClO_4_·3H_2_O, 10 mM LiI, and 1 mM I_2_, at a scan rate of 50 mV·s^−1^ and a potential range of ±1.0 V. The EIS measurements were conducted at a 0 V bias voltage using an amplitude of 5 mV and a frequency range from 100 mHz to 100 KHz in dark conditions. The obtained data was analyzed and fitted by Zview−Version 3.5g software, using the related equivalent circuits as models. Moreover, a 10 mV·s^−1^ scan rate was used to record the Tafel plots. Both the EIS and Tafel samples consisted of two identical CEs and the liquid electrode (CE/electrolyte/CE). The J–V photocurrent density voltage curves of full DSSCs were finally obtained under AM 1.5 G, 0.1 W.cm^−2^ light radiation intensity, by the Newport solar simulator, USA (450 W Xe source), with a Keithley 2400 source meter.

## 3. Results and Discussions

### 3.1. Characterizations of L and F

**Figure 1:**
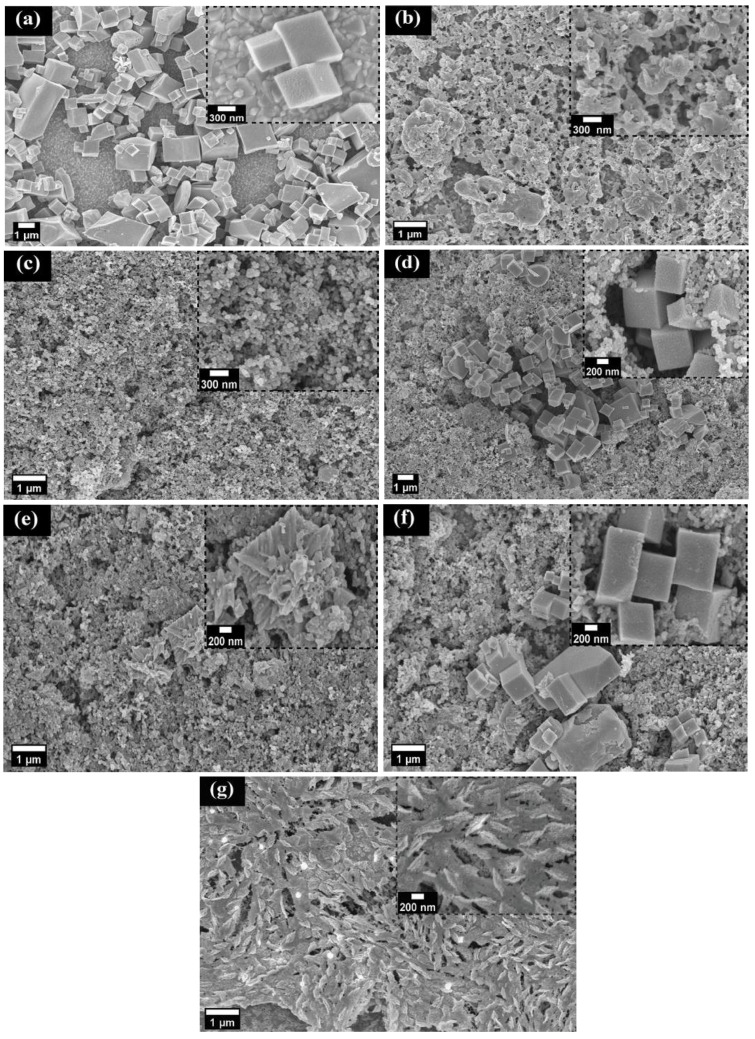
Typical SEM images of FTO/L (**a**), FTO/F (**b**), FTO/CB (**c**), and FTO/L@CB (**d**), FTO/F@CB (**e**), FTO/2-L@F@CB (**f**), and FTO/Pt (**g**).

**Figure 2:**
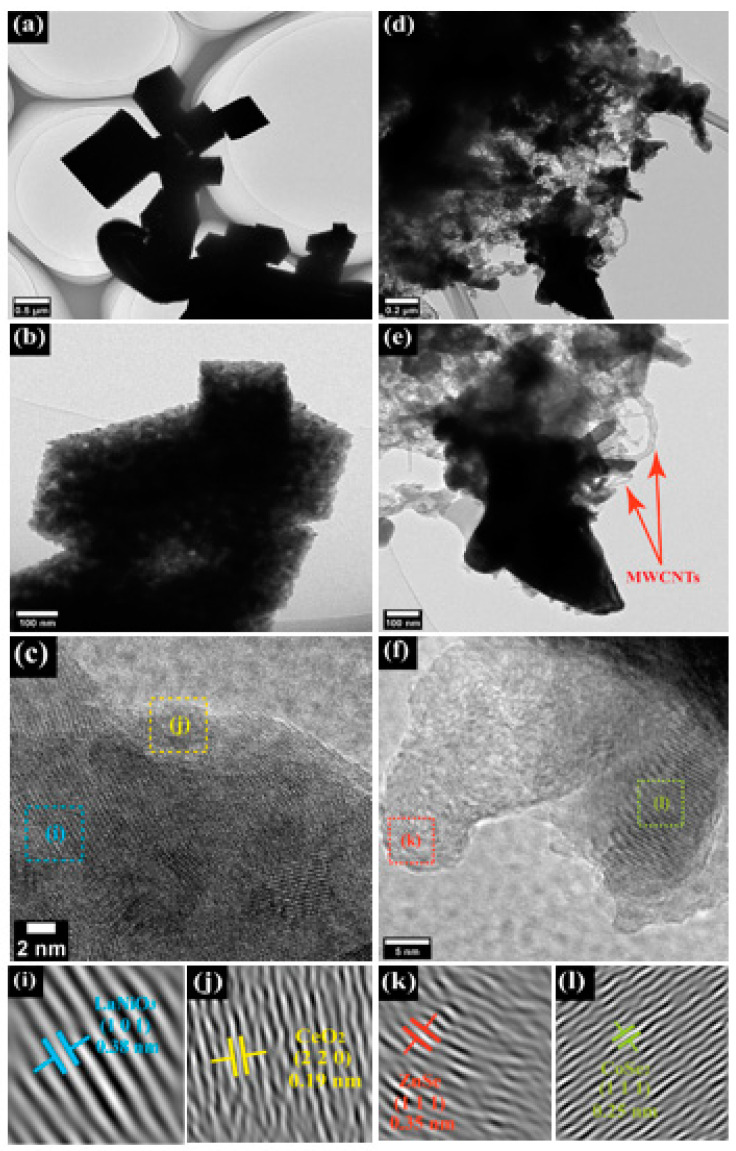
Typical TEM images of L (**a**–**c**,**i**,**j**), and F (**d**–**f**,**k**,**l**).

FESEM was used to analyze the morphology of the prepared CEs. Figure 1a–c shows that L, F, and CB are made of porous nanocubes, amorphous nanoparticles, and round-granular nanoparticles, respectively. This is further confirmed in Figure 1f where the 2-L@F@CB composite also contains three kinds of nanoparticles. We also observed the FESEM cross-sectional images in Figure S1 (ESI) which display good adhesion between FTO glass substrates and the coated nanoparticles. Furthermore, we could observe the average film thicknesses of 0.34 µm, 2.04 µm, 12.21 µm, 3.66 µm, and 0.44 µm for L, F, CB, 2-L@F@CB and Pt, respectively. Therefore, 2-L@F@CB has a thicker film, which generally contributes to better overall performance as CE in DSSCs [32]. The detailed microstructure of L and F was investigated using HRTEM. As displayed in Figure 2, L and F are made of porous nanocubes and amorphous nanoparticles with the presence of MWCNTs, respectively. The lattice fringes in HRTEM show a fringe spacing of 0.38 nm and 0.19 nm (Figure 2i,j), which can be assigned to rhombohedral LiNO_3_ (101) and cubic CeO_2_ (220), respectively. Moreover, the fringes of 0.35 nm and 0.25 nm (Figure 2k,l) can be assigned to cubic ZnSe (111) and orthorhombic CoSe_2_ (111), respectively.

An EDS analysis was performed to evaluate the distribution of different atoms in the L and F nanostructures. The EDS mapping in Figure S2a–f displays well-distributed atoms of La, Ce, Ni, and O over the entire structure of L nanocubes. As for the EDS mapping in Figure S2g–l, it displays a homogeneous distribution over the entire structure of F of Co, Se, and C atoms, but not for Zn. Indeed, the lower melting point (419.5 °C) of Zn metal compared to Co metal (1495.1 °C) gives rise to its phase-separation from other elements during the selenization process, causing the separation of ZnSe in the composite [27].

**Figure 3:**
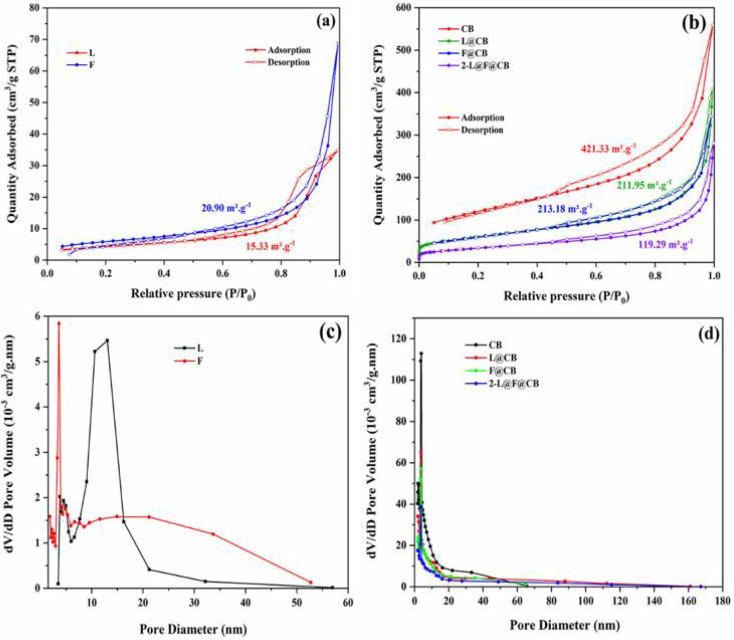
Nitrogen adsorption–desorption isotherms (**a**,**b**), pore size distributions of L, F, CB, L@CB, F@CB and 2-L@F@CB (**c**,**d**).

The BET and BJH were used to assess the surface area and pore size distribution of the nanoparticles. In Figure 3a,b, each prepared sample displays a standard type-IV isotherm with an H3 hysteresis loop at high relative pressure (above P/P_0_ = 0.9). This indicates the mesoporous nature of our samples [33]. Furthermore, the BET surface areas were calculated to be 15.33, 20.90, 421.33, 211.95, 213.18, and 119.29 m^2^. g^−1^, respectively, for L, F, CB, L@CB, F@CB, and 2-L@F@CB (Table 1). We can observe an improvement in the surface areas of F@CB and 2-L@F@CB composites compared to the surface areas of L and F. Moreover, Figure 3c,d shows pore size diameters mostly ranging between 2 and 50 nm for the prepared samples, which confirms their mesoporous structure. High surface area porous materials give rise to higher electrocatalytic activity, which is of benefit for CE applications in DSSCs [34].

XPS was employed to study the nature of chemical bonds and chemical states of L, F, and 2-L@F@CB (Figure S3). The survey spectrum in Figure S3a reveals peaks such as Zn 2p, Ce 3d, Ni 2p, La 3d, Co 2p, O 1s, C 1s, Se 3d, and Co 3p. The spectra of Zn 2p (Figure S3b) display two strong peaks located at ~1044 eV and ~1021 eV, related to the binding energies of Zn 2p_1/2_ and Zn 2p_3/2_, respectively. These two strong peaks are consistent with Zn^2+^ in the binding energies of Zn–Se. [35] The Ce 3d spectra in Figure S3c present for 2-L@F@CB and L, different peaks located at ~915 eV, ~899.7 eV (Ce^3+^: ~904.4 eV, and Sat.: ~896.18 eV), and ~880.4 eV (Sat.: ~886.4 eV). These peaks can be assigned to the binding energies of Ce^4+^ 3d_3/2_, Ce^3+/4+^ 3d_5/2_, and Ce^3+^ 3d_5/2_ found in CeO_x_, respectively [36]. The Ni 2p spectra in Figure S3d display peaks at ~871 eV and ~858.6 eV (Sat.: ~865.4 eV and ~863.9 eV) for 2-L@F@CB, and peaks at ~870.5 eV and ~856.3 eV (Sat. 864.9 eV and 861 eV) for L. In both samples, the two categories of peaks can be ascribed respectively to the characteristics of Ni^2+/3+^ for Ni 2p_1/2_ and Ni 2p_3/2_ occurring in LaNiO_3_ [37]. We can also observe half of the La 3d spectrum in Figure S3d located at ~849.5 eV (Sat. ~853.5 eV) and corresponding to the characteristics of La^3+^ for La 3d_5/2_ occurring in LaNiO_3_ [38]. The La 3d spectra in Figure S3e display peaks located at ~849.5 eV (sat. ~853.5 eV), and ~833 eV (sat. ~837 eV) which can be ascribed to the binding energy of La 3d_5/2_ and La 3d_3/2_, respectively. Both types of peaks are consistent with the results for La^3+^ in a La (III) oxidation state. [38] The spectra of Co 2p (Figure S3f) is made up of two spin–orbit doublets and two shake-up satellites (Sat.). In F and 2-L@F@CB, the peaks located at ~796 eV with shoulders at ~802 eV (Sat.) can be ascribed to Co 2p_1/2_, while the peaks found at ~781 eV with a shoulder at ~785 eV (Sat.) can be assigned to Co 2p_3/2_. Both kinds of peaks match well with the characteristics of Co^2+^ in CoSe_2_ [39]. Furthermore, we can observe in F, two weak peaks at ~793.2 eV and ~776.5 eV which correspond to Co^3+^ of Co 2p_1/2_ and Co 2p_3/2_, respectively. The presence of Co^3+^ peaks in the Co 2p spectrum of this sample is due to the partial surface oxidation under an air atmosphere. However, the main valence state of Co in both samples is the divalent metal cation. As for the shake-up satellites at higher energy, it is due to the antibonding orbital occurring between Se and Co atoms [40]. Figure S3g presents the spectrum of O 1s for 2-L@F@CB and L. The peaks located at ~531 eV, ~529.5 eV, ~528 eV, and ~527 eV correspond respectively to the characteristics of ${\mathrm{OH}}^{-}/O_{2}$, $O_{2}^{2-}$/O, O−La, and O−Ni occurring in LaNiO_3_ [15,16]. The spectra of C 1s (Figure S3h) reveal for F and 2-L@F@CB peaks at around 290 eV, 287 eV, 285.3 eV (shifting to ~283.8 eV in 2-L@F@CB), and 283.9 eV (shifting to ~282.8 eV in 2-L@F@CB) corresponding to O–C=O, C–O, C–C, and C=C bonding, respectively. Furthermore, we can observe at ~282 eV a supplementary peak visible in F and corresponding to C-metal (C-Zn/Co). The peaks related to C=C bonds display quite a high intensity, while those corresponding to C=O bonds show low intensity. This reveals the formation of graphitic carbon during the selenization process of F [27]. In Figure S3i, we can observe for F and 2-L@F@CB, two kinds of deconvoluted peaks at ~55 eV and ~54.3 eV (shifting to 53.2 eV in 2-L@F@CB), matching well with Se 3d_3/2_ and Se 3d_5/2_, respectively. These peaks are both consistent with the metal–Se interaction occurring in CoSe_2_. As for the broad peaks located within 62–57 eV, they are consistent with Se–O bonding on the surface, and Co 3p [27]. Therefore, the 2-L@F@CB composite is made up of the same chemical bonds as those observed in L and F, with very slight peak shifts due to changes in the chemical environment. Moreover, we could observe in 2-L@F@CB lower peak intensities compared to L and F for almost all peaks except for C 1s (especially C=C) and O 1s (especially $O_{2}^{2-}$/O). The increase in the intensity of C=C can be attributed to the graphitic carbon-rich environment favored by F and CB in the 2-L@F@CB composite. Moreover, the increase in the intensity of $O_{2}^{2-}$/O (Figure S4) in 2-L@F@CB can be ascribed to the contribution of L to increasing the oxygen vacancy percentage, made possible thanks to the replacement of La sites with Ce in its structure. Abundant oxygen vacancies lead to more active sites, giving rise to minimized efficiency losses in DSSCs [17,21].

Figure S5a,b presents the XRD patterns of L, F, CB, L@CB, F@CB, and 2-L@F@CB. The diffraction peaks located at 25.0°, 32.8°, 47.5°, and 58.8° can be assigned, respectively, to (101), (110), (202), and (300) crystal planes of rhombohedral LiNO_3_ (JCPDS No. 00-034-1077). We can also observe in 2-L@F@CB that the diffraction peaks located at 32.8°, and 47.5° can be related to (303) and (611) lattice planes observed in monoclinic La_2_O_3_ (JCPDS No. 00-022-0641). These sharp diffraction peaks located at 32.8° and 47.5° can also be respectively ascribed to (200) and (220) crystal planes of cubic CeO_2_ (JCPDS No. 01-081-0792). As for the diffraction peaks located at 37.0° and 58.8°, they correspond respectively to the (101) and (107) crystal planes of rhombohedral NiO_2_ (JCPDS No. 01-085-1977). The sharp diffraction peaks located at 30.8°, 34.6°, 36.0°, 47.8°, and 57.0° can be respectively ascribed to (110), (111), (012), (121), and (211) lattice planes of orthorhombic CoSe_2_ (JCPDS No. 00-010-0408). The diffraction peaks located at 27.5° and 45.4° correspond to (111) and (220) crystal planes of cubic ZnSe (JCPDS No. 01-080-0021). As for the diffraction peaks located at 37.3°, 44.1°, 50.4°, and 53.5° they can be assigned to (0012), (114), (0016), and (1110) lattice planes of orthorhombic carbon (JCPDS No. 01-074-2330). Furthermore, the diffraction peaks located at 25.0°, and 43.2° can be ascribed to the (002) and (101) crystal planes of hexagonal graphite carbon (JCPDS No. 01-075-1621). These patterns indicate that LaNiO_3_ and CoSe_2_ with high purity were formed respectively during the synthesis of perovskite oxide and the selenization process. The XRD pattern of 2-L@F@CB confirms that it is a new composite prepared from the synthesized L and F, which did not display any obvious peak shifts in the pattern.

### 3.2. Measurements of Electrochemical Properties of CEs

**Figure 4:**
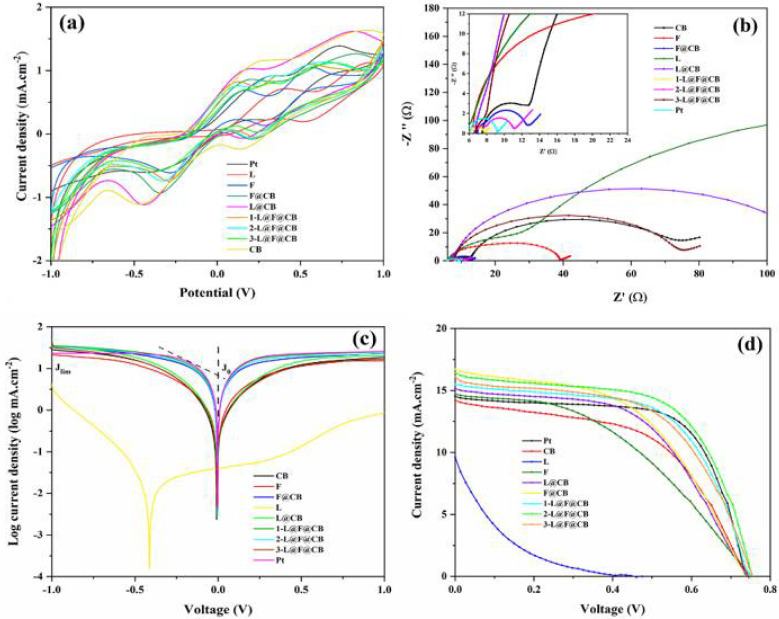
CV plots of various CEs at 50 mV.s^−1^ (**a**); EIS Nyquist plots (**b**); Tafel polarization plots (**c**); J–V plots of DSSCs using Pt, CB, L, F, L@CB, F@CB, 1-L@F@CB, 2-L@F@CB, and 3-L@F@CB based CEs (**d**).

We then assessed the electrocatalytic activities of various CEs of Pt, L, F, CB, L@CB, F@CB, 1-L@F@CB, 2-L@F@CB, and 3-L@F@CB for triiodide ion ($I_{3}^{-}$) reduction by the CV technique (Figure 4a). For each sample, we can see two pairs of standard oxidation–reduction current density peaks, respectively named (Ox1, Red1) for the lower potential region related to the chemical reaction in Equation (1, and (Ox2, Red2) for the high potential region corresponding to the chemical reaction in Equation (2).

(1) $$I_{3}^{-}+2e^{-}↔3I^{-}$$

(2) $$3I_{2}+2e^{-}↔2I_{3}^{-}\;$$

The redox peaks located at a more negative potential allow assessment of the electrocatalytic performance and can be assigned to the chemical reaction of Equation (1) [41]. Therefore, we examined the electrocatalytic parameters such as peak-to-peak separation (∆E_pp_) and reduction peak current density (J_Red1_) only for the low potential region. ∆E_pp_ is directly linked to the kinetic redox capability of the CE in the $I^{-}/I_{3}^{-}$ redox couple. The smaller the ∆E_pp_ value, the higher the electrocatalytic ability of the corresponding CE [42]. Table 2 summarizes the main CV parameters obtained for our different CEs. ∆E_pp_ values show the trend of 1-L@F@CB (389 mV) < F (413 mV) < 2-L@F@CB (414 mV) < 3-L@F@CB (470 mV) < L (480 mV) < F@CB (511 mV) < Pt (604 mV) < L@CB (607 mV) < CB (647 mV). In this trend, L@F@CB composites show higher electrocatalytic activities, which can be related to the higher electrocatalytic of both F composites and L, and the excellent electrical conductivity of CB [43]. We can also observe for the reduction peak current density (|J_Red1_|) the following trend of L@CB (1.104 mA·cm^−2^) > CB (1.073 mA·cm^−2^) > F@CB (0.921 mA·cm^−2^) > 1-L@F@CB (0.735 mA·cm^−2^) > 2-L@F@CB (0.730 mA·cm^−2^) > F (0.606 mA·cm^−2^) > 3-L@F@CB (0.561 mA·cm^−2^) > Pt (0.551 mA·cm^−2^) > L (0.004 mA·cm^−2^). These values suggest that L@F@CB composites have high reduction ability, in good agreement with the results of ∆E_pp_. Furthermore, based on the trends of ∆E_pp_ and |J_Red1_|, it can be concluded that 1-L@F@CB and 2-L@F@CB demonstrate better electrocatalytic activity than Pt. Moreover, we assessed the ratio J_Ox1_/|J_Red1_| for the low potential region, very relevant for the catalytic activity and gives information about the reversibility of the redox reaction [27]. We can see from Table 2 that the J_Ox1_/|J_Red1_| for 2-L@F@CB is closer to 1 than all the other samples, including Pt (1.624), indicating a more reversible reaction for 2-L@F@CB [44].

We also analyzed, for the 2-L@F@CB-based CE, the CV plots obtained for various scan rates and noticed that increasing the scanning speed leads to an increase in the anodic and cathodic peak current densities. Consequently, we found a direct link between the peak current densities and the square root of the scan rate, which means that no chemical reaction occurs between the redox couple and the 2-L@F@CB-based CE (Figure S6a,b). Furthermore, a direct connection indicates that the cathodic/anodic reactions have restricted the diffusion of the flow of iodide species in the cell toward the CE. This results from the narrowing of the diffusion layer occurring with the increase of the scanning speed and the electrochemical polarization, giving rise to high over-potential and reduced reversibility [27]. In addition, we also investigated the electrochemical stability of 2-L@F@CB-based CE through consecutive CV measurements at the scan rate of 50 mV.s^−1^ (Figure S6c,d). Indeed, electrochemical stability is highly important for the potential application of the CE in DSSCs. Even after 100 cycles, no obvious change was observed in the morphology of J_Red1_ and ∆E_pp_. The stability of the 2-L@F@CB-based CE is the result of the high surface area and good conductivity of F, L, and CB. Furthermore, the binding properties of CB contribute to the strong adhesion of this CE.

An EIS was also carried out to deeply study the electrocatalytic activity of CEs from symmetric dummy cells made of two identical CEs. Figure 4b presents the Nyquist plots for our CEs. We can notice that Pt, F, and L_-_based CEs show two semicircles, while CB, F@CB, L@CB and all L@F@CB-based CEs display three semicircles, resulting from their porous nature [27]. In standard Nyquist plots, the series resistance (R_s_) is the intercept of the first semicircle with the horizontal axis; the charge-transfer resistance (R_ct_) and the capacitance of the CE/electrolyte interface are linked to the first semicircle (high frequency region). As for the middle semicircle (related to the middle frequency), it is linked to the adsorption of iodine. Finally, the last semicircle (low frequency region) is related to the Nernst diffusion impedance of the triiodide/iodide redox couple within a thin layer of the electrolyte (W) [27]. We obtained the EIS data by fitting the Nyquist plots with an equivalent circuit (Figure S7), and the related data are listed in Table 2. The R_s_ values for F, F@CB, L@CB, and L@F@CB-based CEs are all closer to the R_s_ value for Pt CE than the R_s_ values for L and CB CEs. Furthermore, the R_ct_ values display the trend of 1-L@F@CB (0.685 Ω) < 2-L@F@CB (0.848 Ω) < F@CB (1.096 Ω) < 3-L@F@CB (1.194 Ω) < L@CB (1.470 Ω) < Pt (3.004 Ω) < CB (4.996 Ω) < F (12.550 Ω) < L (16.170 Ω). We can observe that upon the addition of F and L to CB, the R_ct_ value strongly decreases gradually from 12.550 Ω, 16.170 Ω and 4.996 Ω, respectively, for F, L, and CB, to 0.685 Ω and 0.848 Ω for 1-L@F@CB and 2-L@F@CB. These values are significantly lower than the 3.004 Ω obtained for Pt CE. Indeed, the lower the R_ct_ value, the faster the electron transfer from the surface of the CE to the electrolyte, leading to less interfacial recombination [45]. Therefore, 1-L@F@CB and 2-L@F@CB have the best catalytic activity among our CEs, in confirmation of the previous CV analysis.

Tafel polarization was also investigated to determine the catalytic activity of various CEs. A typical Tafel polarization plot has three regions: the polarization region (|V| < 120 mV), the Tafel region (120 mV < |V| < 400 mV), and the diffusion region (|V| > 400 mV). For investigation of the catalytic performance of various CEs, we studied the diffusion region. The corresponding two key parameters, namely the exchange current density (J_0_) and the limiting diffusion current density (J_lim_), are connected to the catalytic ability of each sample and can be obtained, respectively, from the Tafel and diffusion regions. Moreover, J_0_ and J_lim_ can also be obtained by calculation using Equations (3) and (4) [27].

(3) $$J_{0}=\frac{\mathrm{RT}}{\mathrm{nFRct}}$$

(4) $$J_{\lim}=\frac{2\mathrm{nFCDn}}{l}$$

where R stands for the gas constant, T for the temperature in Kelvin, F for the Faraday constant, n for the number of electrons transferred per chemical reaction between the electrolyte and the CE interface (n = 2), Dn for the diffusion coefficient of $I_{3}^{-}$, l for the thickness of the diffusion layer, and C for the concentration of $I_{3}^{-}$. Moreover, Dn can be further obtained from the Randles–Sevcik theory [46], (Equation (5), with $J_{\mathrm{red}}$ corresponding to the peak current density, $K=2.69\times 10^{5}$, A being the active area of the symmetric cell, and υ representing the applied scan rate of CV plots.

(5) $$J_{\mathrm{red}}={\mathrm{Kn}}^{1.5}{\mathrm{ACD}}_{n}^{0.5}\upsilon^{0.5}$$

In Table 2, we can see a noticeable increase in the J_0_ values after the mixing of L and F into CB. These values increase from 0.094, 7.656 and 9.484 mA·cm^−2^ for L, F, and CB, respectively, to 23.768 and 22.646 mA·cm^−2^ for 1-L@F@CB and 2-L@F@CB, respectively. Both values are superior to the J_0_ value of 19.275 mA·cm^−2^ obtained for the Pt-based CE. In addition, the small increase in the J_0_ value observed from 2-L@F@CB (22.646 mA·cm^−2^) to 1-L@F@CB (23.768 mA·cm^−2^) indicates that 2-L@F@CB is the best CE as mentioned in the previous CV investigation. Moreover, Table 2 displays the Dn values in the decreasing order of L@CB (4.211 × 10^−7^ cm^−2^·s^−1^) > CB (3.978 × 10^−7^ cm^−2^·s^−1^) > F@CB (2.931 × 10^−7^ cm^−2^·s^−1^) > 1-L@F@CB (1.866 × 10^−7^ cm^−2^·s^−1^) > 2-L@F@CB (1.841 × 10^−7^ cm^−2^·s^−1^) > F (1.269 × 10^−7^ cm^−2^·s^−1^) > 3-L@F@CB (1.087 × 10^−7^ cm^−2^·s^−1^) > Pt (1.049 × 10^−7^ cm^−2^·s^−1^) > L (5.528 × 10^−12^ cm^−2^·s^−1^). We can see the high Dn values of 1-L@F@CB (1.866 × 10^−7^ cm^−2^·s^−1^), and 2-L@F@CB (1.841 × 10^−7^ cm^−2^·s^−1^) CEs compared to Pt (1.049 × 10^−7^ cm^−2^·s^−1^) CE. This indicates the high velocity of these materials, which is advantageous for improving the photovoltaic performance of the DSSCs. 

Table 3 presents the photovoltaic parameters of various CEs in DSSCs, and the corresponding J–V plots are visible in Figure 4d. We can observe that when used separately as CEs for DSSCs, F and L allow the achievement of PCEs of 4.73% and 0.42%, respectively. These values were significantly improved with the L@F@CB composites. Indeed, when properly adjusting the mass ratio (1.5:3:1.5) of L:F:CB, the PCE of the corresponding DSSC (2-L@F@CB) significantly increases, up to 7.49%. This value is superior to the value obtained for the Pt-based DSSC (7.09%) counterpart, leading to better electrocatalytic activity in the composite. These outcomes were first favored by the increase in the oxygen vacancy percentage offered by L thanks to the replacement of La sites with Ce. This gives rise to more active sites in the final L@F@CB composite. Secondly, this high electrocatalytic activity and good performance result from the joint benefits of L, F, and CB holding high electrocatalytic activities. Moreover, this achievement is justified by the high electrical conductivity of the carbon-based materials created by the F@CB composite, which boosts the conductivity with the contribution of the hybrid CNT@CB [23]. Finally, the strong adhesion of CB with FTO glass contributes, in agreement with the above CV stability test results, to the stability of the 2-L@F@CB based DSSC. All this leads to lower R_ct_ and lower diffusion resistance in the 2-L@F@CB based CE, confirming the previous morphological and electrochemical analysis. However, the lowering of PCE observed in the 3-L@F@CB composite-based DSSC is the result of the agglomeration of particles in the composite, giving rise to reduced performance.

### 3.3. Device Stability

**Figure 5:**
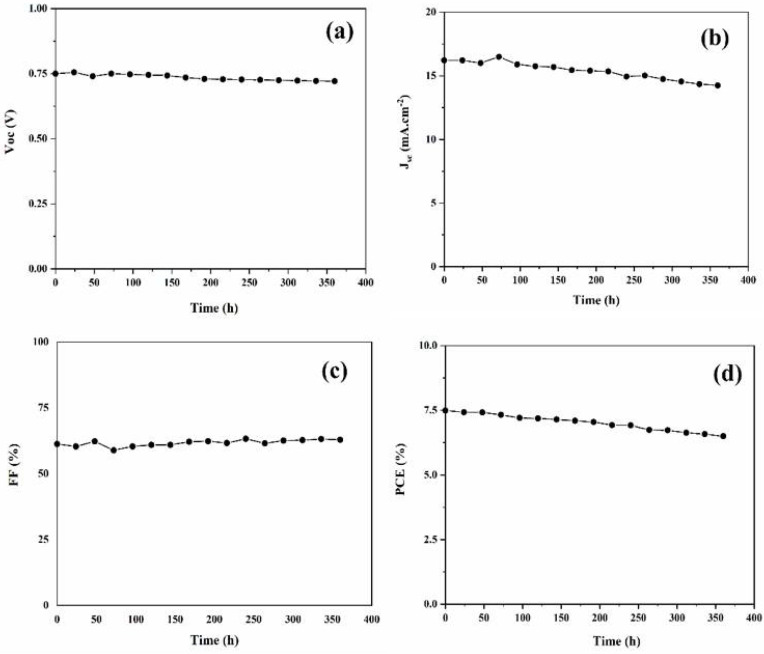
Device stability test under illumination for DSSC made of 2-L@F@CB CE. (**a**) Voc, (**b**) Jsc, (**c**) FF, and (**d**) PCE.

We also examined for 360 h (15 days) the device stability of the DSSC made of 2-L@F@CB CE. The DSSC was kept in dark conditions after each IV measurement, and the photovoltaic performance was tested under 100 mA cm^−2^ illumination at regular intervals of time. Figure 5 shows the photovoltaic parameters recorded for the analyzed time frame. At the 360th h (15th day), the DSSC shows a PCE of 6.50% with 14.25 mA·cm^−2^ (Jsc), 0.721 V (Voc), and 62.93 (FF). This result indicates about a 13% decrease in the PCE after 360 h of testing, which is quite good performance under laboratory conditions. These results also confirm the performance of the 2-L@F@CB CE for DSSCs.

### 3.4. Effect of 2-L@F@CB Film Thickness on DSSCs’ Performance

**Figure 6:**
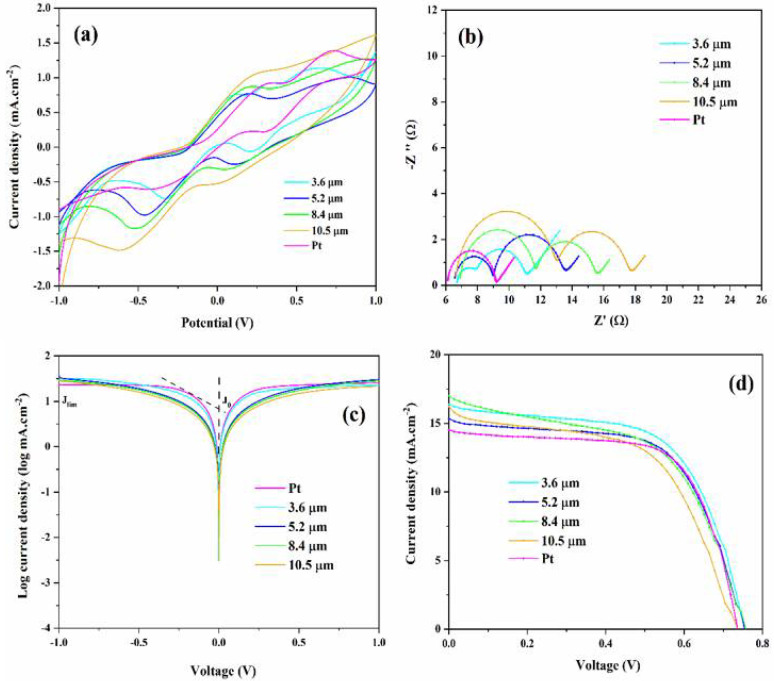
CV plots at 50 mV·s^−1^ scan rate (**a**); EIS Nyquist plots (**b**); Tafel polarization plots (**c**); DSSC J–V plots (**d**) of 2-L@F@CB CEs with different film thicknesses.

2-L@F@CB composite CEs with various film thicknesses ranging from 3.6 to 10.4 μm were prepared to investigate their effects on DSSCs’ performance. We also prepared Pt CE for comparison. Figure 6a shows the CV curves of 2-L@F@CB CEs with different film thicknesses. As displayed in Table S1, the peak current density ranges from 0.730 to 1.470 mA·cm^−2^, with the corresponding peak-to-peak separation also increasing from 414 to 787 mV, respectively, for 3.6 and 10.4 μm thickness. These results suggest that despite the increase in surface area, thicker 2-L@F@CB films give rise to lower catalytic activity, which can be due to the increase in internal resistance [25,47]. We also recorded the EIS data for 2-L@F@CB CEs with the various film thicknesses. Figure 6b shows the Nyquist plots with the corresponding parameters listed in Table S1**.** There is no obvious variation in the Rs values for all 2-L@F@CB CEs with different thicknesses. However, there is a significant increase in the R_ct_ values from 0.848 to 6.357 Ω, respectively, for 3.6 and 10.4 μm film thickness. This increase in R_ct_ resistance indicates a significant lowering of the electrocatalytic activity with the increase of 2-L@F@CB film thickness, in agreement with the previous CV analysis. Tafel polarization curves were also analyzed as shown in Figure 6c, and the resultant parameters are listed in Table S1. The J_o_ and J_lim_ values of the 2-L@F@CB CEs greatly decrease with the increase in film thickness. These results indicate an increase in the diffusion resistance of the redox couple in the electrolyte with 2-L@F@CB film thickness. Therefore, the DSSCs made of 2-L@F@CB CEs with thinner films are expected to show optimum performance comparable or higher than Pt-based cells. We finally investigated the effect of 2-L@F@CB CE film thicknesses on the performance of DSSCs as shown in Figure 6d, and the corresponding photovoltaic parameters are reported in Table S2**.** We could observe a slight decrease of about 12% (from 7.49% to 6.55%) in the PCE with the increase in film thickness from 3.6 to 10.4 μm. This outcome can be attributed to the slight lowering in J_SC_ and FF values resulting from the increase in internal resistance as demonstrated in the previous analysis. Therefore, the optimum efficiency was achieved for DSSCs prepared with a 3.6 μm film thickness.

## 4. Discussion on the Potential Cost of Prepared CEs’ Materials

In this section, we compare the potential prices of the prepared CEs’ materials. Although the price per gram of Pt is always fluctuating because of widespread demand, it is generally high as given in Table S3. In Figure S8a, we can observe the very low cost of the materials involved in our work compared to Pt, and this leads to L@F@CB composites being cheaper than Pt, with a cost-saving percentage of more than 30% (Figure S8b). This further confirms the potential of prepared L@F@CB CEs over Pt CE.

## 5. Conclusions

L perovskite oxide, F, and L@F@CB composites have been prepared with success and analyzed for the first time as CEs for DSSCs. L perovskite oxide was synthesized using the GNP method, while F was obtained from the hydrothermal selenization of f-MWCNT/ZIF-8@ZIF-67. The electrochemical analysis reveals that the optimized L@F@CB CE exhibits better electrocatalytic activity than the Pt CE. Moreover, the related DSSCs achieved a superior PCE of 7.49% than the corresponding Pt-based DSSC (7.09%) prepared under similar conditions. The enhanced performance is due to the increase in the oxygen vacancy percentage offered by L thanks to the replacement of La sites with Ce. This gives rise to more active sites in the final L@F@CB composite. Moreover, the F@CB composite favors the contribution to the high electrical conductivity of the hybrid carbon nanotube–carbon black (CNT@CB), which also offers stability to the L@F@CB CE. Consequently, the corresponding L@F@CB-based device achieved enhanced stability. Our work demonstrates that L@F@CB composites with a low cost are excellent alternatives to Pt CE in DSSCs.

## Figures and Tables

**Table 1:** BET and BJH parameters for L, F, CB, L@CB, F@CB, and 2-L@F@CB.

CEs	BET (m^2^·g^−1^)	BJH Desorption Cumulative Surface Area of Pores (m^2^·g^−1^)	BJH Desorption Cumulative Volume of Pores (cm^3^·g^−1^)	BJH Desorption Average Pore Diameter (4V/A) (nm)
L	15.33	18.45	0.05	11.73
F	20.90	24.53	0.11	17.25
CB	421.33	394.46	0.84	8.56
L@CB	211.95	231.42	0.63	10.95
F@CB	213.18	203.65	0.52	10.26
2-L@F@CB	119.29	137.54	0.44	12.67

**Table 2:** CV, EIS, and Tafel polarization parameters for different CEs.

CEs	∆E_pp_ (mV)	J_Ox1_ (mA·cm^−2^)	│J_Red1_│ (mA·cm^−2^)	J_Ox1_ /│J_Red1_│	R_s_ (Ω)	R_ct-EIS_ (Ω)	J_0_ (mA·cm^−2^)	J_lim_ (mA·cm^−2^)	Dn (cm^−2^·s^−1^)
Pt	604	0.895	0.551	1.624	6.113	3.004	19.275	21.878	1.04891 × 10^−7^
CB	647	1.115	1.073	1.039	7.494	4.996	9.484	11.951	3.97773 × 10^−7^
F	413	0.680	0.606	1.122	6.441	12.550	7.656	9.376	1.26876 × 10^−7^
L	480	0.711	0.004	177.75	5.492	16.170	0.094	0.003	5.52784 × 10^−12^
F@CB	511	0.848	0.921	0.921	6.897	1.096	17.258	19.143	2.93059 × 10^−7^
L@CB	607	1.022	1.104	0.926	6.589	1.470	11.117	14.060	4.21089 × 10^−7^
1-L@F@CB	389	0.821	0.735	1.117	6.071	0.685	23.768	22.336	1.86642 × 10^−7^
2-L@F@CB	414	0.754	0.730	1.033	6.833	0.848	22.646	20.559	1.84112 × 10^−7^
3-L@F@CB	470	0.687	0.561	1.225	6.730	1.194	7.691	11.940	1.08733 × 10^−7^

**Table 3:** Photovoltaic performance of DSSCs with different CEs under AM 1.5 G illumination at 100 mW·cm^−2^.

CE	V_OC_/V	J_SC_/mA·cm^−2^	FF%	PCE/%
Pt	0.74	14.41	66.79	7.09
CB	0.74	14.08	53.35	5.58
L	0.46	9.61	9.44	0.42
F	0.75	14.64	43.29	4.73
L@CB	0.74	15.02	53.68	5.99
F@CB	0.74	16.59	50.47	6.16
1-L@F@CB	0.75	15.42	60.47	6.97
2-L@F@CB	0.75	16.22	61.27	7.49
3-L@F@CB	0.76	15.78	56.44	6.73

## Data Availability

Data can be available upon request from the authors.

## Supplementary Materials

The following supporting information can be downloaded at: https://www.mdpi.com/article/10.3390/nano12060961/s1, Figure S1: FESEM Cross-sectional images of L (a), F (b), CB (c), 2-L@F@CB (d), and Pt (e), Figure S2: EDS mapping of L (a, b, c, d, e, f), and F (g, h, i, j, k, l), Figure S3: XPS spectra of porous L, F and 2-L@F@CB: (a) Survey XPS spectra; (b) Zn 2p XPS spectra; (c) Ce 3d XPS spectra; (d) Ni 2p and La 3d XPS spectra; (e) La 3d XPS spectra; (f) Co 2p XPS spectra; (g) O 1s XPS spectra; (h) C 1s XPS spectra; and (i) Se 3d and Co 3p XPS spectra, Figure S4: Percentage of Oxygen Vacancy in L and 2-L@F@CB, Figure S5: XRD patterns of (a) L, F, CB; and (b) L@CB, F@CB, 2-L@F@CB. (♥) LiNO_3_ perovskite oxide, (#) La_2_O_3_, (ѱ) CeO_2_, (○) NiO_2_, (●) CoSe_2_, (♣) ZnSe, (♦) C-Orthorhombic, and (⁎) C Graphite-Hexagonal, Figure S6: (a) CV curves of 2-L@F@CB CE at different scan rates; (b) Corresponding relationship between peak current densities and the square root of scan rates of 2-L@F@CB CE; (c) 100 times consecutive CV curves of 2-L@F@CB CE at the 50 mV·s^−1^ scan rate in iodine based electrolyte; (d) Corresponding ΔEpp values for the 100 times consecutive CV curves of 2-L@F@CB CE, Figure S7: Equivalent circuit models for: (a) Pt, F and L symmetric cells; (b) CB, F@CB, L@CB and all L@F@CB composites based symmetric cells, Table S1: CV, EIS, and Tafel polarization parameters for 2-L@F@CB CEs with various film thicknesses, Table S2: Photovoltaic performance of DSSCs for 2-L@F@CB CEs with various film thicknesses under AM 1.5G illumination at 100 mW·cm^−1^, Figure S8: Comparison of Different Compounds’ Prices: (a) Main Elements, (b) CEs’ Materials, Table S3: Comparison of CEs’ Materials Prices based on Different Product Sources.
