# Supplementary material for: Enhanced Performance of Carbon–Selenide Composite with La0.9Ce0.1NiO3 Perovskite Oxide for Outstanding Counter Electrodes in Platinum-Free Dye-Sensitized Solar Cells
Source: Nanomaterials (Basel). 2022 Mar 14;12(6):961. doi: 10.3390/nano12060961 (PMC8953699; doi:10.3390/nano12060961)
Supplement: Supplementary file 1 [file nanomaterials-12-00961-s001.zip › nanomaterials-1565989-supplementary.pdf]

Supplementary Materials

Enhanced Performance of Carbon–Selenide Composite with $La_{0.9}Ce_{0.1}NiO_3$ Perovskite Oxide for Outstanding Counter Electrodes in Platinum-Free Dye-Sensitized Solar Cells

Arnauld Robert Tapa [1,2], Wanchun Xiang [1,3,*], Senwei Wu [1], Bin Li [1], Qiufen Liu [1], Mingfeng Zhang [1], Marzieh Ghadamyari [4], Francis Verpoort [4,5], Jichao Wang [4], Albert Trokourey [2] and Xiujian Zhao [1,*]

1 State Key Laboratory of Silicate Materials for Architecture, Wuhan University of Technology, Luoshi Road, Wuhan 430070, China; tapaarnauld@yahoo.fr (A.R.T.); senwei_wu@whut.edu.cn (S.W.); libin625@whut.edu.cn (B.L.); qiufen.liu@whut.edu.cn (Q.L.); 15084978457@163.com (M.Z.)

2 Laboratory of Constitution and Reaction of Matter, Training and Research Unit for Structural Sciences of Matter and Technology, Félix Houphouët-Boigny University of Cocody-Abidjan, Abidjan 22 BP 582, Côte d'Ivoire; trokourey@gmail.com

3 Key Laboratory for Applied Surface and Colloid Chemistry, Ministry of Education, Shaanxi Key Laboratory for Advanced Energy Devices, Shaanxi Engineering Laboratory for Advanced Energy Technology, School of Materials Science & Engineering, Shaanxi Normal University, Xi'an 710119, China

4 Laboratory of Organometallics, Catalysis and Ordered Materials, State Key Laboratory of Advanced Technology for Materials Synthesis and Processing, Wuhan University of Technology, Wuhan 430070, China; marzieh.ghadamyari@yahoo.com (M.G.); francis.verpoort@ghent.ac.kr (F.V.); wangjichao5475@163.com (J.W.)

5 National Research Tomsk Polytechnic University, Lenin Avenue 30, 634050 Tomsk, Russia

* Correspondence: xiangwanchun@whut.edu.cn (W.X.); opluse@whut.edu.cn (X.Z.)

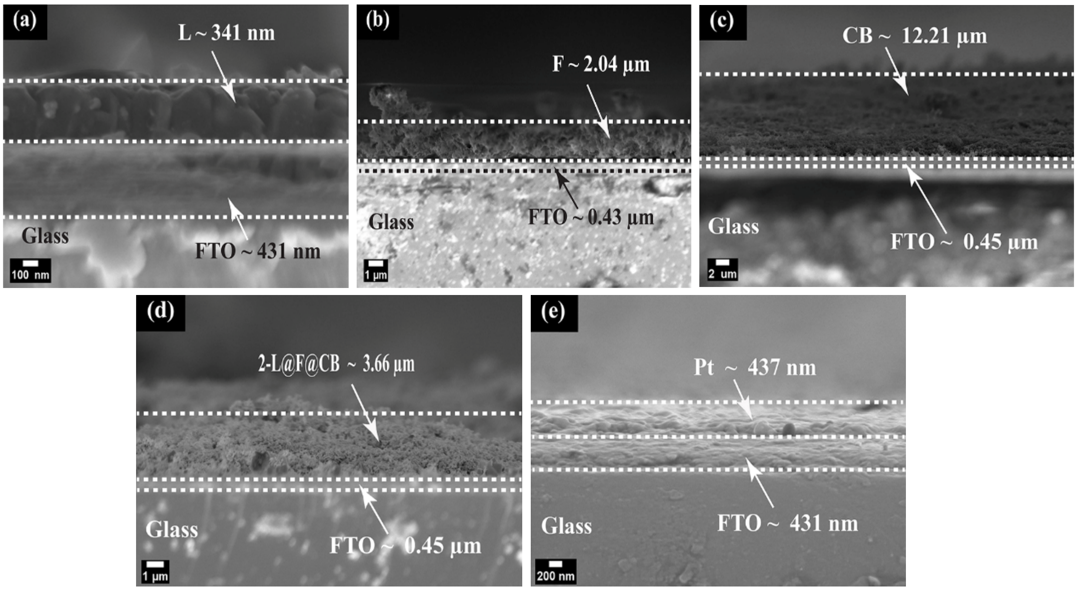

Figure S1. FESEM Cross-sectional images of L (a), F (b), CB (c), 2-L@F@CB (d), and Pt (e).

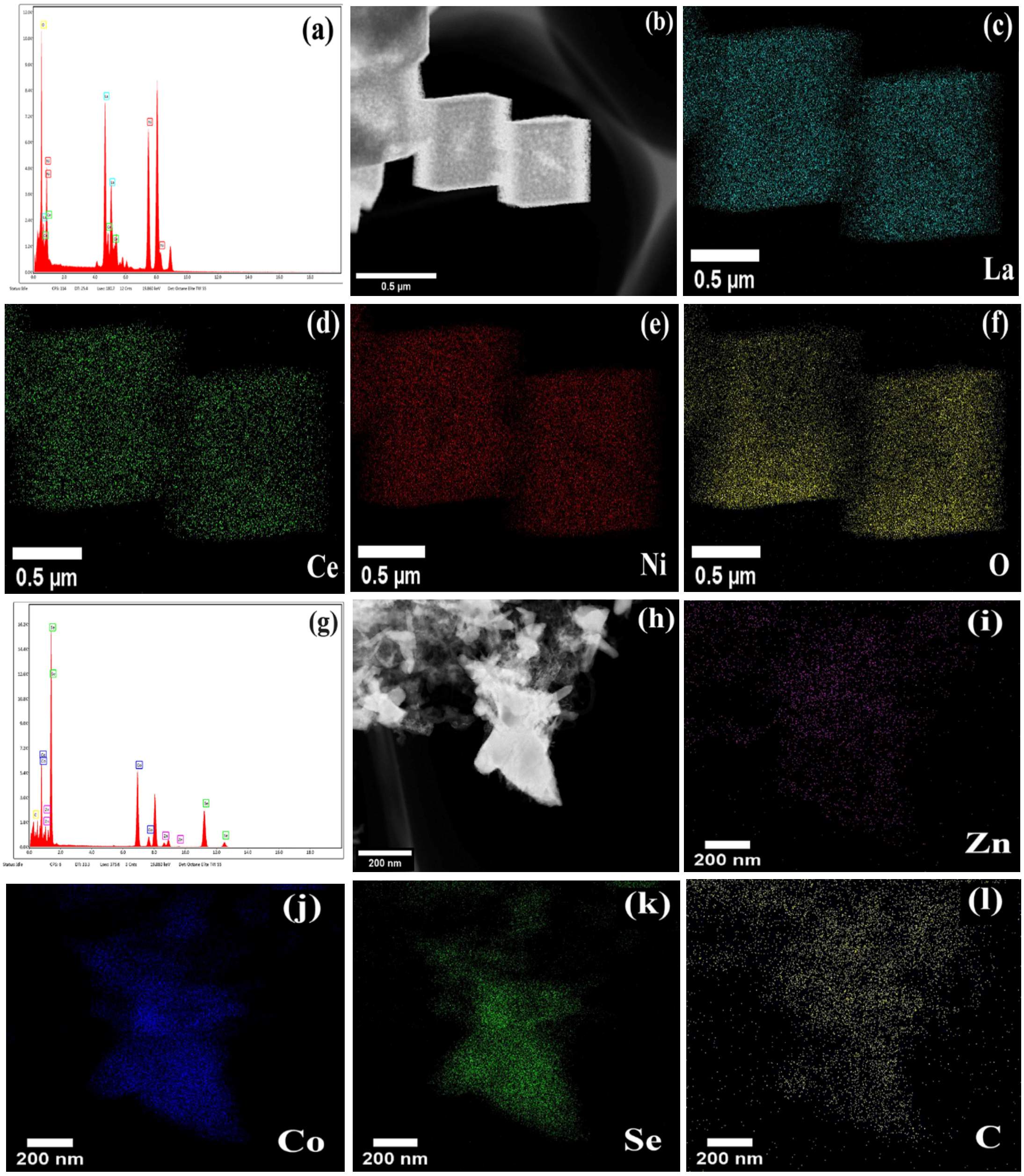

Figure S2. EDS mapping of L (a, b, c, d, e, f), and F (g, h, i, j, k, l).

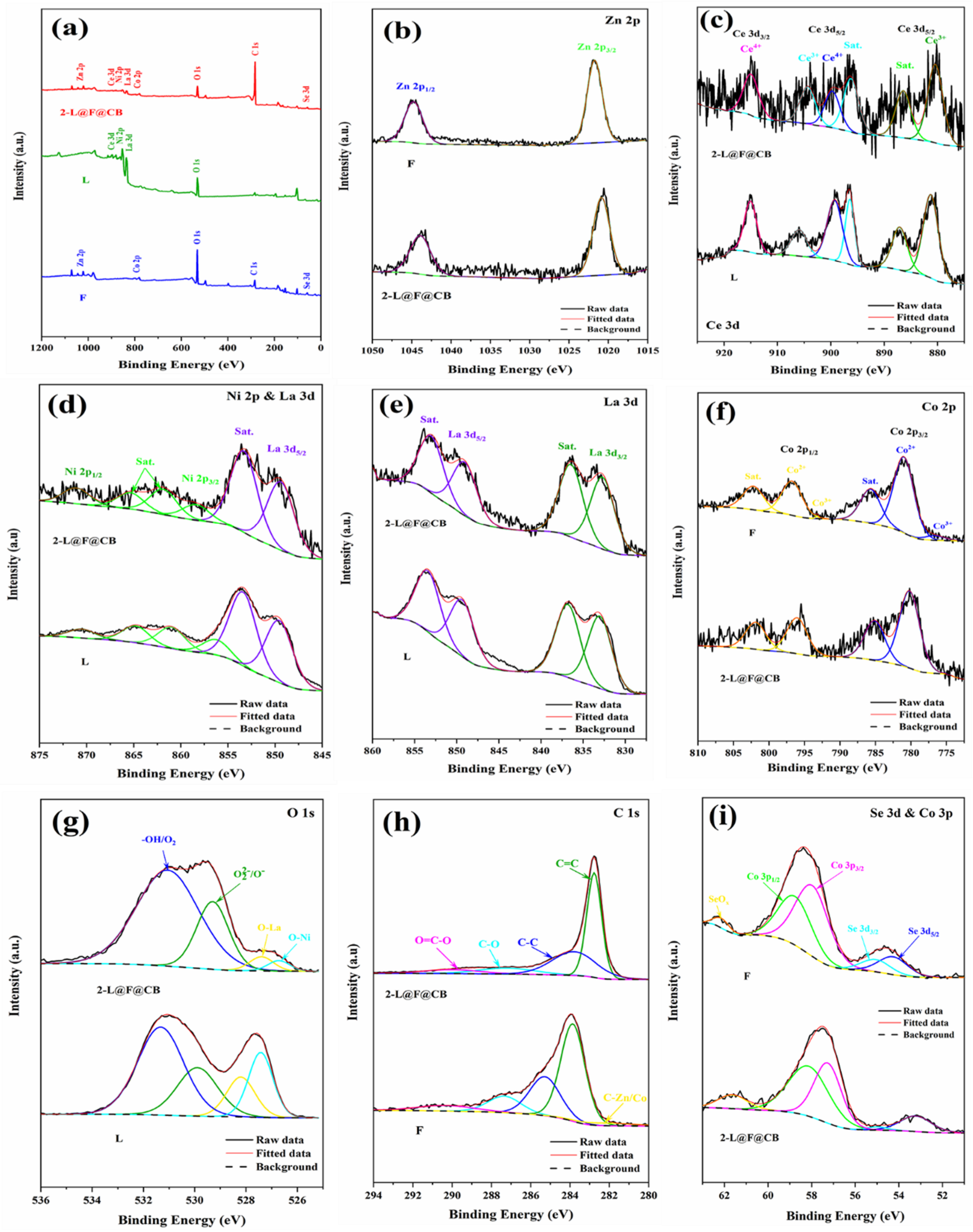

Figure S3. XPS spectra of porous L, F and 2-L@F@CB: (a) Survey XPS spectra; (b) Zn 2p XPS spectra; (c) Ce 3d XPS spectra; (d) Ni 2p and La 3d XPS spectra; (e) La 3d XPS spectra; (f) Co 2p XPS spectra; (g) O 1s XPS spectra; (h) C 1s XPS spectra; and (i) Se 3d and Co 3p XPS spectra.

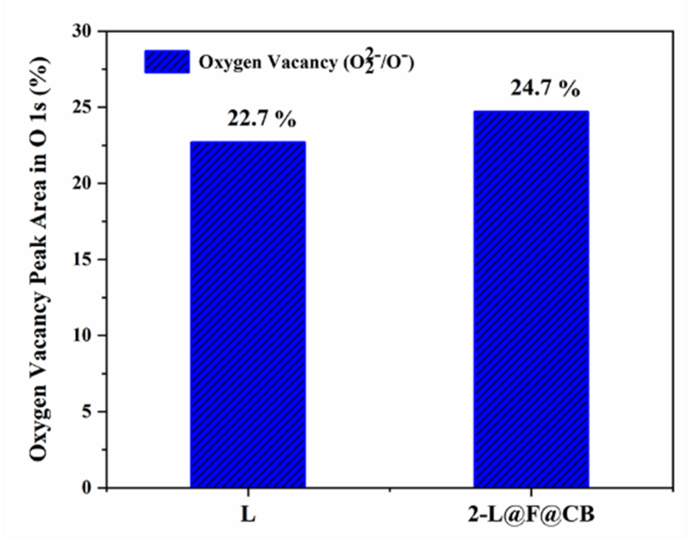

Figure S4. Percentage of Oxygen Vacancy in L and 2-L@F@CB.

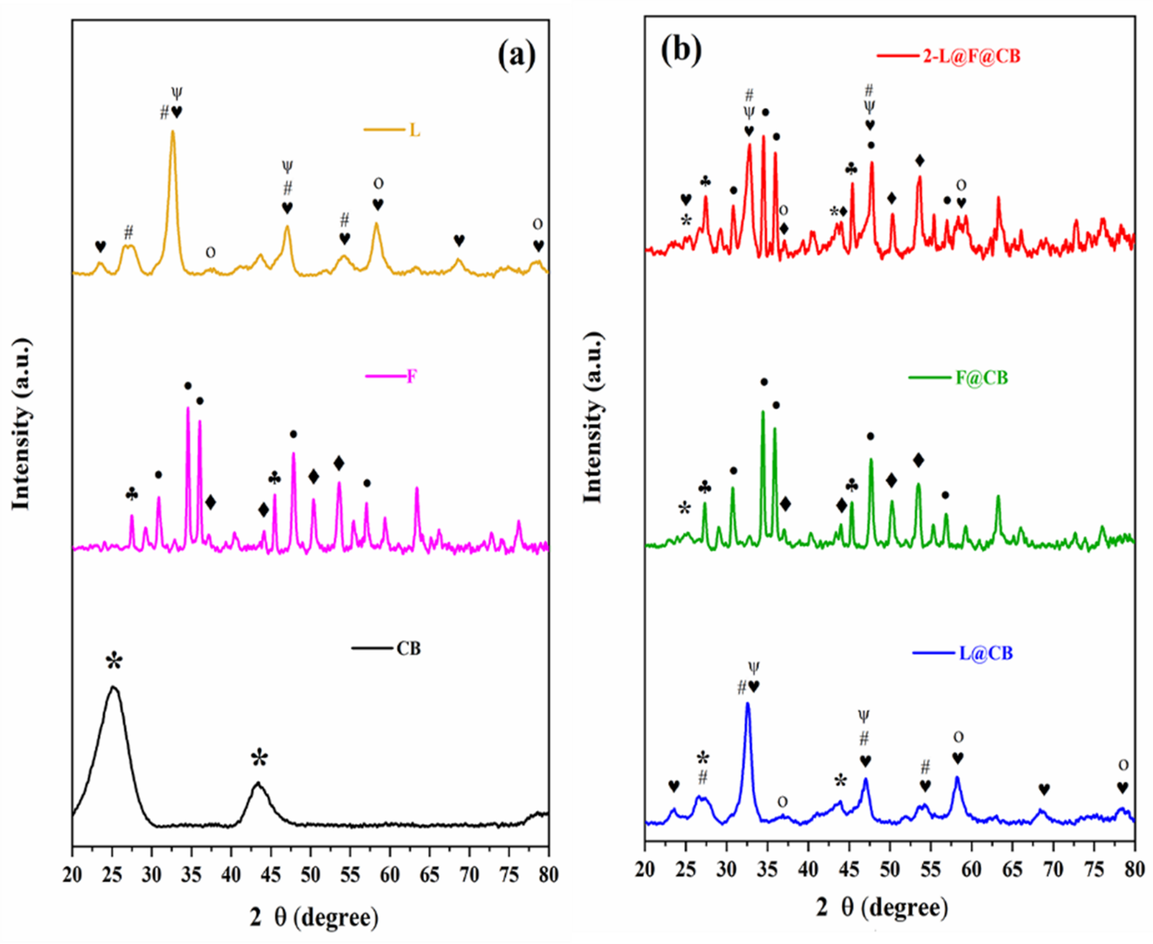

Figure S5. XRD patterns of (a) L, F, CB; and (b) L@CB, F@CB, 2-L@F@CB. (♥) $LiNO_3$ perovskite oxide, (#) La_2O_3, (ψ) CeO_2, (○) NiO_2, (●) $CoSe_2$, (♣) ZnSe, (♦) C-Orthorhombic, and (*) C Graphite-Hexagonal.

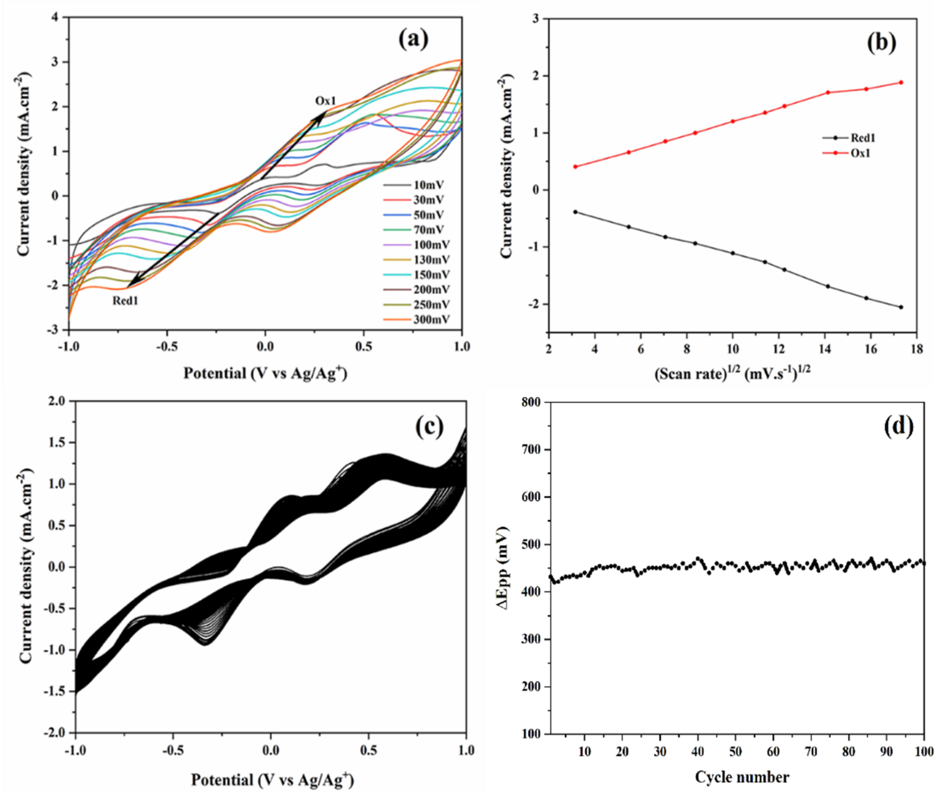

Figure S6. (a) CV curves of 2-L@F@CB CE at different scan rates; (b) Corresponding relationship between peak current densities and the square root of scan rates of 2-L@F@CB CE; (c) 100 times consecutive CV curves of 2-L@F@CB CE at the 50 mV·s^{-1} scan rate in iodine based electrolyte; (d) Corresponding ΔEpp values for the 100 times consecutive CV curves of 2-L@F@CB CE.

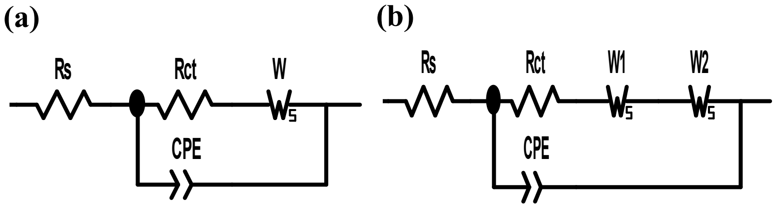

Figure S7. Equivalent circuit models for: (a) Pt, F and L symmetric cells; (b) CB, F@CB, L@CB and all L@F@CB composites based symmetric cells.

Table S1. CV, EIS, and Tafel polarization parameters for 2-L@F@CB CEs with various film thicknesses.

CE	ΔE_{pp} (mV)	J_{Ox1} (mA.cm^{-2})	$\lvert J_{Red1} \rvert$ (mA.cm^{-2})	J_{Ox1} / $\lvert J_{Red1} \rvert$	R_s (Ω)	$R_{ct\text{-}EIS}$ (Ω)	J_0 (mA.cm^{-2})	J_{lim} (mA.cm^{-2})
Pt (0.44 μm)	604	0.895	0.551	1.624	6.113	3.004	19.275	21.878
3.6 μm	414	0.754	0.730	1.033	6.833	0.848	22.646	20.559
5.2 μm	616	0.757	0.972	0.779	6.534	2.46	16.59	15.49
8.4 μm	678	0.857	1.162	0.737	6.894	4.784	15.49	14.45
10.4 μm	787	1.012	1.470	0.688	6.623	6.357	13.80	12.88

* The thickness for Pt CE was obtained from cross sectional SEM image (**Figure S1**)

Table S2. Photovoltaic performance of DSSCs for 2-L@F@CB CEs with various film thicknesses under AM 1.5G illumination at 100 mW·cm^{-2}

CE	V_{OC}/V	J_{SC}/mA.cm^{-2}	FF%	PCE/%
Pt (0.44 μm)	0.74	14.41	66.79	7.09
3.6 μm	0.75	16.22	61.27	7.49
5.2 μm	0.75	15.15	62.61	7.14
8.4 μm	0.76	16.84	55.01	7.01
10.4 μm	0.73	15.77	56.73	6.55

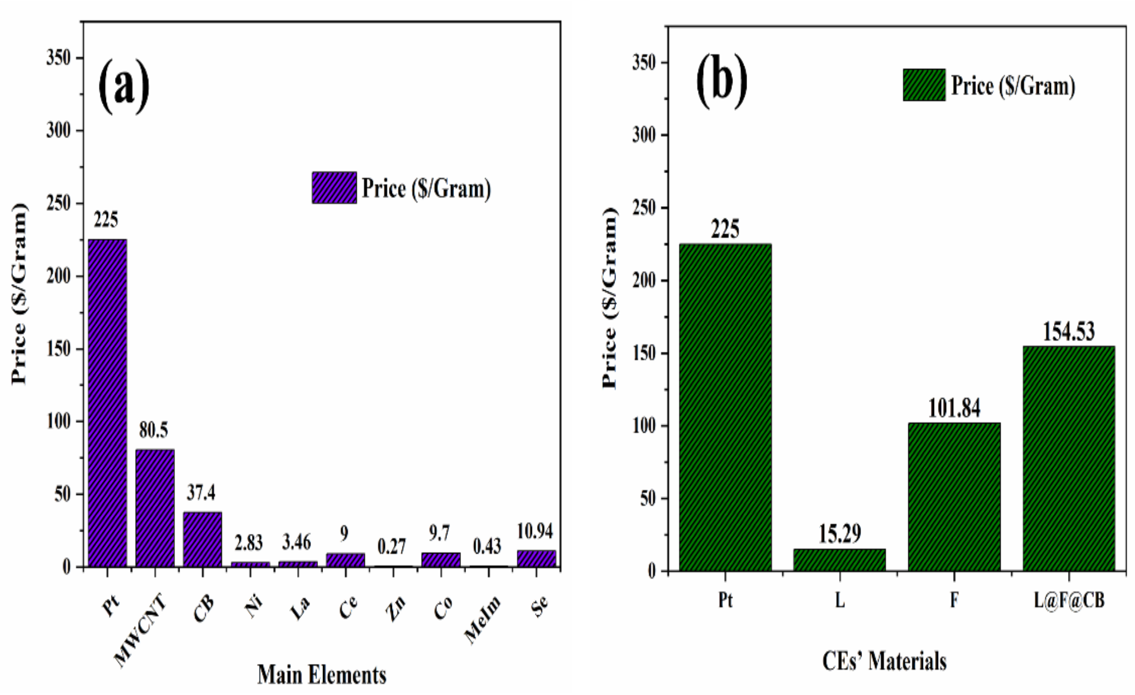

Figure S8. Comparison of Different Compounds' Prices: (a) Main Elements, (b) CEs' Materials

Table S3. Comparison of CEs' Materials Prices based on Different Product Sources (Sigma Aldrich)

**CEs' Materials**	**Product Sources**	**Main Elements**	**Price ($/Gram)**	**Total Price ($/Gram)**
**Pt**	H_2PtCl_6	Pt	$ 225.00 / g	**$ 225.00 / g**
L	$La(NO_3)_3{\cdot}6H_2O$	La	$ 3.46 / g	$ 15.29 / g
	$Ce(NO_3)_3{\cdot}6H_2O$	Ce	$ 9 / g	
	$Ni(NO_3)_2{\cdot}6H_2O$	Ni	$ 2.83 / g	
F	$Zn(NO_3)_2{\cdot}6H_2O$	Zn	$ 0.27 / g	$ 101.84 / g
	$CoCl_2{\cdot}6H_2O$	Co	$ 9.7 / g	
	Selenium powder	Se	$ 10.94 / g	
	2-methylimidazole	MeIm	$ 0.43 / g	
	MWCNT	C	$ 80.5 / g	
CB	CB	C	$ 37.4 / g	$ 37.4 / g
**L@F@CB**				**$ 154.53 / g**
